# Tanshinones, Critical Pharmacological Components in *Salvia miltiorrhiza*

**DOI:** 10.3389/fphar.2019.00202

**Published:** 2019-03-14

**Authors:** Zhouqian Jiang, Wei Gao, Luqi Huang

**Affiliations:** ^1^School of Traditional Chinese Medicine, Capital Medical University, Beijing, China; ^2^School of Pharmaceutical Sciences, Capital Medical University, Beijing, China; ^3^State Key Laboratory of Dao-di Herbs, National Resource Center for Chinese Materia Medica, China Academy of Chinese Medical Sciences, Beijing, China

**Keywords:** tanshinones, *Salvia miltiorrhiza*, extraction and separation, pharmacological activities, biosynthesis

## Abstract

*Salvia miltiorrhiza* Bunge, a member of the Lamiaceae family, is valued in traditional Chinese Medicine. Its dried root (named Danshen) has been used for hundreds of years, primarily for the treatment of cardiovascular and cerebrovascular diseases. Tanshinones are the main active ingredients in *S. miltiorrhiza* and exhibit significant pharmacological activities, such as antioxidant activity, anti-inflammatory activity, cardiovascular effects, and antitumor activity. Danshen dripping pill of Tianshili is an effective drug widely used in the clinical treatment of cardiovascular diseases. With the increasing demand for clinical drugs, the traditional method for extracting and separating tanshinones from medicinal plants is insufficient. Therefore, in combination with synthetic biological methods and strategies, it is necessary to analyze the biosynthetic pathway of tanshinones and construct high-yield functional bacteria to obtain tanshinones. Moreover, the biosynthesis of tanshinones has been studied for more than two decades but remains to be completely elucidated. This review will systematically present the composition, extraction and separation, pharmacological activities and biosynthesis of tanshinones from *S. miltiorrhiza*, with the intent to provide references for studies on other terpenoid bioactive components of traditional Chinese medicines and to provide new research strategies for the sustainable development of traditional Chinese medicine resources.

## Introduction

*Salvia miltiorrhiza* Bunge, an important Salvia species with terrific economic, social and medicinal benefits (Su et al., 2015), was first recorded in the *Shenlong Bencao Jing* (200–300 AD, Han Dynasty), the oldest medicine monograph in China. *Salviae miltiorrhizae* Radix et Rhizoma, dry roots of Danshen, has been widely applied in the clinical treatment of cardiovascular diseases (Chen and Chen, 2017), dysmenorrhea, amenorrhea, and hypertension, hepatocirrhosis, chronic renal failure and other diseases (Su et al., 2015). With the development of science and technology, the dosage forms containing Danshen have been gradually diversified. Tablet, injection solution, dripping pill, oral liquid, capsule, slow-release formulation, and soft gel are all dosage forms that have been prepared into medicines. Among these diverse preparations, composite Danshen droplet pills, used to treat angina pectoris and coronary heart disease, represent a star drug and, moreover, a demonstration of traditional Chinese medicine entering the international market. Currently, advanced clinical tests on this drug are ongoing in the United States. The active ingredients in *S. miltiorrhiza* (Table 1) can be mainly divided into two categories: fat-soluble tanshinone compounds and water-soluble salvianolic acids. Both ingredients have significant pharmacological activities and are also representative active ingredients in traditional Chinese medicine (Ma et al., 2015), especially tanshinones.

**Table 1:** A list of abbreviations would be adequate to define all abbreviations used in the study.

Abbreviation	Full name
*S. miltiorrhiza*	Salvia miltiorrhiza Bunge
SFE	Supercritical fluid extraction
CUAE-HIUP	Continuous ultrasound-assisted extraction with high intensity ultrasonic probe
SPE	Solid-phase extraction
HPLC	High performance liquid chromatography
HSCCC	Semi-preparative high-speed countercurrent chromatography
DNA	Deoxyribonucleic acid
NADPH	Nicotinamide adenine dinucleotide phosphate
ROS	Reactive oxygen species
STS	Sodium tanshinone IIA silate
VSMC	Vascular smooth musle cell
AMPK	Adenosine 5′-monophosphate (AMP)-activated protein kinase
MI	Myocardial infarction
ox-LDL	Oxidized low density lipoprotein
AD	Alzheimer’s disease
BBB	Blood-brain barrier
iNOS	Inducible nitric oxide synthase
IPP	Ispentenyl diphosphate
DMAPP	Dimethylallyl diphosphate
MEP	2-*C*-methyl-D-erythritol 4-phosphate
MVA	Mevalonate
IDS	Isoprenyl diphosphate synthase
GGPP	Geranylgeranyl diphosphate
TPS	Terpene synthases/cylases
HMGR	3-Hydroxy-3-methylglutaryl-CoA reductase
MeJA	Methyl jasmonate
AACT	Acetyl-CoA C-acyltransferase
HMGS	3-Hydroxy-3-methylglutaryl-coenzyme A synthase
MK	Mevalonate kinase
PMK	Phosphomevalonate kinase
MDC	Mevalonate diphosphate decarboxylase
Pyr	Pyruvate
G3P	Glyceraldehyde 3-phosphate
DXS	1-Deoxy-D-xylulose-5-phosphate synthase
DXP	1-Deoxy-D-xylulose 5- phosphate
DXR	1-Deoxy-D-xylulose 5- phosphate reductoisomerase
IDI	Isopentenyl diphosphate isomerase
GPPS	Geranyl diphosphate synthase
FPPS	Farnesyl diphosphate synthase
GGPPS	Geranylgeranyl diphosphate synthase
RACE	Rapid amplification of cDNA ends
CPS	Labdadienyl/Copalyl diphosphate synthase
KSL	Kaurene synthase-like
GA	Gibberellin
CYP	Cytochrome p450
CPR	Cytochrome p450 reductase
UV-B	Ultraviolet-B

As secondary metabolites, tanshinones mainly accumulate in the roots, but the yield is very low. Currently, tanshinones are obtained from roots by chemical separation and purification. However, because traditional methods have low efficiency, high energy consumption and unfriendliness to the environment and plant resources, it is meaningful and necessary to find new methods. With the successful applications of synthetic biology technology in the study of taxol, ginsenoside, and artemisinin (Huang et al., 2014), this approach could also provide new developments and approaches for the modern study of tanshinones (Shi et al., 2018). Based on research on tanshinones at home and abroad, this paper will systematically review the research results of extraction, purification, pharmacological activity and biosynthesis of the original plant *S. miltiorrhiza*.

## The Plant *S. miltiorrhiza*

*Salvia miltiorrhiza* Bunge belongs to the genus Salvia of the Lamiaceae family, which is a perennial erect herb. Its dry root is used as Danshen and has the effects of promoting blood circulation, relieving pain, regulating heat, and calming the nerves.

Wild *S. miltiorrhiza* generally grows in central and northeastern China. Environmental and soil conditions affect the quality of Danshen and the content of active ingredients (Peng et al., 2014). The Chinese Flora reports that there are two varieties in the *S. miltiorrhiza* species, namely, the original variety (*S. miltiorrhiza* var. *miltiorrhiza*) and *S. miltiorrhiza* Bunge var. *charbonnelii*. The original variety can be divided into two forms, namely, the original form (*S. miltiorrhiza* var. *miltiorrhiza* f. *miltiorrhiza*) and *S. miltiorrhiza* var. *miltiorrhiza* f. *alba*. These forms mainly differ in plant morphology and geographical distribution. For example, the flower color of *S. miltiorrhiza* var. *miltiorrhiza* f. *alba* is white, and it originates from Shandong, China.

In recent years, due to the excessive harvesting of wild *S. miltiorrhiza*, the resources are already on the verge of extinction. With the reduction in wild resources, artificial domestication cultivation of *S. miltiorrhiza* has been carried out since the 1970s. The main producing areas of the current cultivated *S. miltiorrhiza* are Linyi Shandong Province, Jiaozuo Henan Province, Wanrong Shanxi Province, Shangluo Shaanxi Province, Zhongjiang Sichuan Province and other places. To date, *S. miltiorrhiza* from Sichuan Province is still considered to have the best quality. Due to the low yield of secondary metabolites and the long growth cycle of cultivated plants, the production of tanshinones from cultivated *S. miltiorrhiza* can no longer meet the rapidly growing market demand. It is essential to use modern biotechnology methods to increase the yield. Therefore, various *in vitro* culture systems of *S. miltiorrhiza*, including suspension cell (Zhao et al., 2010), callus (Wu et al., 2003), adventitious root and hairy root, as well as new techniques such as using endophytic fungi (Li et al., 2016; Zhou et al., 2018) and transgenic plants (Liu et al., 2017; Wei et al., 2018), can be used to accumulate tanshinone production.

## Tanshinones of *S. miltiorrhiza*

**Figure 1:**
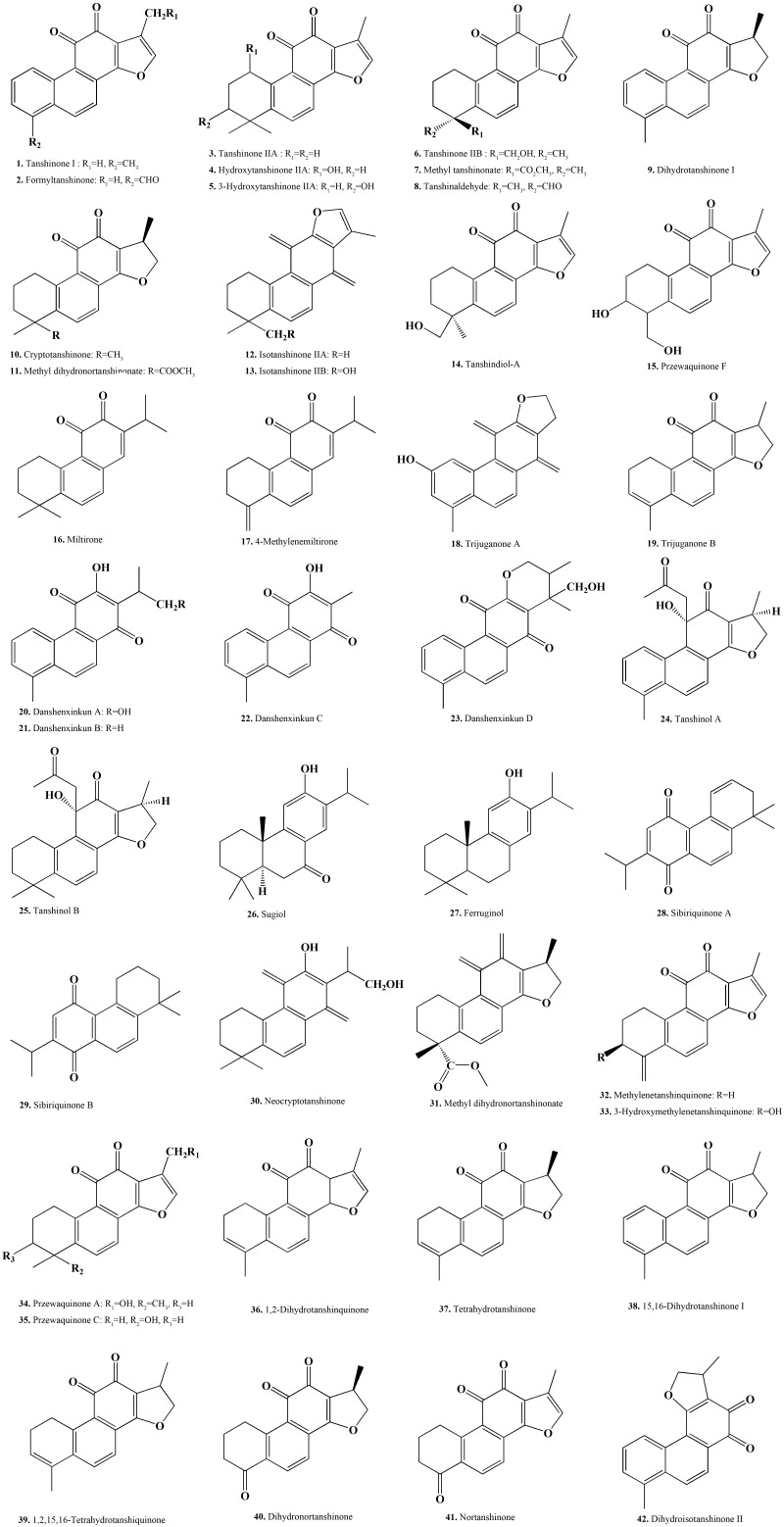
Chemical structure of representative tanshinone compounds contained in *S. miltiorrhiza*.

In the 1930s, domestic and foreign scholars have begun to study the chemical constituents of *S. miltiorrhiza* (Xu et al., 2018). Japanese scholar Nakao first isolated tanshinone IIA from *S. miltiorrhiza* and identified its chemical structure in 1934. After the 1970s, with the development of clinical application of *S. miltiorrhiza* and the advancement of Chinese medicine extraction and separation technology, its chemical composition research has developed rapidly (Kakisawa et al., 1968, 1969). Tanshinones, also known as total tanshinone, are a general term for a class of lipophilic phenanthrene compounds that are rich in the roots of *S. miltiorrhiza* (Ma et al., 2015). What these compounds have in common is that they possess an *ortho-* or *fluorene* structure and can be reduced to a diphenol derivative, which is converted to hydrazine after oxidation. In this process, these molecules facilitate electron transfer, and can promote or interfere with various reactions of the body; accordingly, these compounds exhibit significant pharmacological properties such as anti-cancer, antibacterial and anti-viral effects. To date, more than 40 tanshinones (Pang et al., 2016) have been isolated from *S. miltiorrhiza*, including *ortho*-quinones (i.e., tanshinone I, tanshinone IIA, dihydrotanshinone, and cryptotanshinone) and *para*-quinones (i.e., isotanshinone I, isotanshinone IIA, and isocryptotanshinone). In addition, other diterpenoids and triterpenoids have been isolated from Danshen (Wu et al., 2016), such as danshenxinkun A/B/C/D and neo-tanshinlactone (Gao S. et al., 2012). With the increasing clinical demand for tanshinones, more research is currently being conducted to explore how to efficiently obtain tanshinones and enhance their pharmacological effects by exploring structure activity relationships (Pickens et al., 2011; Krivoruchko and Nielsen, 2015; Luo et al., 2015; Figure 1).

## Extraction and Separation Methods of Tanshinones

Due to the clinical application of tanshinones, the extraction, and purification processes of these compounds were developed early in tanshinone research, and the research on associated extraction and separation process technologies is relatively extensive. The main extraction methods include alcohol extraction, ultrasonic extraction, supercritical CO_2_ fluid extraction (Wang L. et al., 2015), pressurized liquid extraction (PLE) (Ong and Len, 2004; Li et al., 2007), microwave-assisted extraction (Pan et al., 2001), high-speed countercurrent chromatography, column chromatography, and vacuum liquid phase extraction.

Because terpenoids generally have low polarity, they are often extracted with chloroform, ethyl acetate or petroleum ether. For some slightly more polar compounds, the plant roots are usually first degreased and then extracted with a polar solvent such as acetone (Wang et al., 2012) and n-butanol (Pang et al., 2016). Although the alcohol extraction method is a relatively traditional extraction process, it is still widely used in the market for its simple and easy operation, and modern ideas have been incorporated to optimize the method. For example, by using orthogonal design, the best extraction and purification processes for total tanshinones were identified. The content of total tanshinones in crude extract increased from 18.23 to 57.29% by purification.

The ultrasonic extraction method does not require heating due to the short production cycle, thus avoiding the thermal degradation reaction of tanshinone IIA. Compared with traditional methods, such as methanol reflux extraction and organic solvents macerating at room temperature, ultrasonic-assisted extraction and microwave-assisted extraction of tanshinones have advantages such as extraction speed and high efficiency (Pan et al., 2002). Similarly, supercritical fluid extraction technology (SFE) (Wang L. et al., 2015) has a short production cycle and does not require heating, which avoids the degradation reaction of tanshinone IIA. Compared with the traditional alcohol extraction method, SFE can retain more tanshinone IIA, but due to the expensive equipment and high production cost, it still needs further promotion to enter the market.

In addition, the extraction of tanshinones has many new methods and new technologies, such as non-ionic surfactant-assisted extraction (Bi et al., 2011), infrared-assisted extraction (Chen et al., 2010), ionic liquid-based ultrahigh pressure extraction (Liu et al., 2013), and selectively modified microfluidic chips (Xuan et al., 2010). The combined application of methods can make extraction more efficient and productive, for example, ultrasound-assisted extraction combined with an amino-modified monolithic cartridge used as a solid-phase extraction sorbent to improve extraction efficiency (Tao et al., 2013); on-line continuous sampling combined with ionic liquid-based dynamic microwave-assisted extraction (Gao S. et al., 2015); a dynamic continuous ultrasound-assisted extraction with a high-intensity ultrasonic probe (CUAE-HIUP) combined with solid-phase extraction (SPE) (Qiao et al., 2007); and ionic liquid surfactant-mediated ultrasonic-assisted extraction combined with high performance liquid chromatography (HPLC) (Wu et al., 2015).

**Figure 2:**
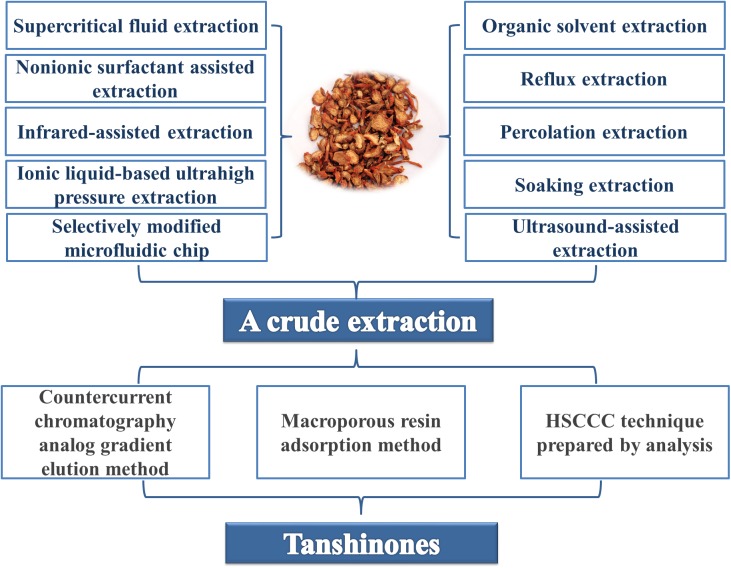
A brief summary of the extraction and separation of tanshinones from the original plants of *S. miltiorrhiza*.

Chromatography is commonly used for the separation and purification of tanshinone monomers. The semipreparative high-speed countercurrent chromatography (HSCCC) technique (Sun et al., 2011) could successfully separate and purify the four major components of Danshen at high purities of over 95% (Tian et al., 2002). Furthermore, the high-efficiency separation of tanshinones could be achieved by the macroporous resin adsorption method and the countercurrent chromatography analog gradient elution method (Wu S. et al., 2012; Figure 2).

## Pharmacological Activities of Tanshinones

**Figure 3:**
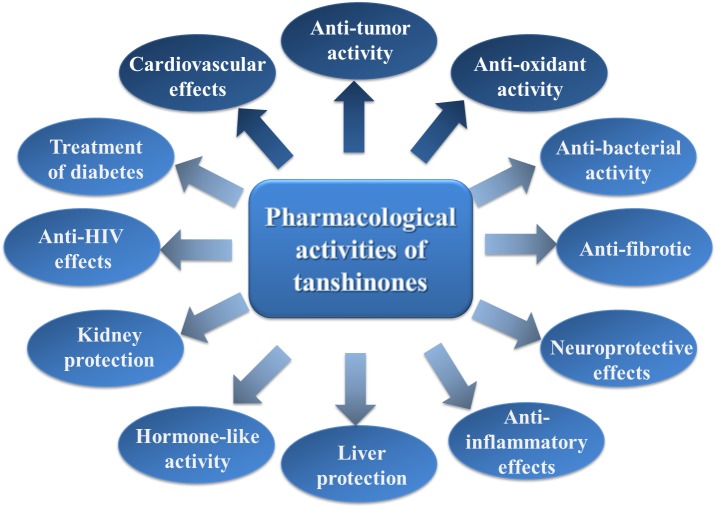
The brief summary of the pharmacological activities of tanshinones. The boxes filled with dark blue indicate that the pharmacological activities are stronger, and the boxes filled with light blue indicate that the pharmacological activities are general.

Tanshinones have received extensive attention due to their remarkable activities in the clinical treatment of cardiovascular diseases. With deepening research, these compounds have been found to possess a wide range of pharmacological activities, such as antibacterial, antioxidant, anti-inflammatory, and anti-tumor properties. A brief introduction to the pharmacological activity of tanshinones will follow (Figure 3).

### Anti-tumor Activity

There are many kinds of anti-tumor drugs, and their mechanisms of action are also different. For example, some drugs can interfere with the anabolism of nucleic acids, inhibit mitosis, and inhibit protein synthesis. Currently, most of the applied chemotherapy drugs are cytotoxic and cannot only kill cancer cells, but also cause side effects (Yuan et al., 2003). The phenanthrene-quinone structure widely present in tanshinone compounds is a major cause of the cytotoxicity (Wu et al., 1991; Shi et al., 1997; Wang et al., 2004). During the antitumor reaction, the DNA molecule binds to the phenanthrene ring structure of the tanshinone, and the furan ring and steroid structure generate free radicals to hinder the synthesis of DNA in the tumor cells. Tanshinone IIA can regulate oxidative stress and reduce the mitochondrial membrane potential to induce apoptosis in cancer cells such as human lung cancer A549 cells (Chiu and Su, 2010); tanshinones inhibit the growth of human prostate cancer cell lines by arresting the cell cycle and inducing apoptosis *in vitro*. Among them, tanshinone I has the most significant activity with IC50 approximately 3–6 μM and its side effects on normal prostate epithelial cells are relatively small (Gong et al., 2011); cryptotanshinone induces tumor cell apoptosis by triggering cell cycle arrest, altering mitochondrial signaling pathways, and by other apoptosis-regulating proteins or death receptor pathways; the methanol extract of *S. miltiorrhiza* induced apoptosis in non-small cell lung cancer through phosphatase and tensin homolog (PTEN)-mediated inhibition of the PI3K/Alct pathway (Ye et al., 2017); *S. miltiorrhiza* exerted clear cytotoxic effects, and strongly inhibited the proliferation of HepG2 cells (Liu et al., 2000); and tanshinone I inhibits tumor angiogenesis by the phosphorylation of reducing signal transducers and activators of transcription (STAT3) at TYR705 and hypoxia-induced HIF-1a accumulation in both endothelial and tumor cells (Wang Y. et al., 2015). In summary, tanshinones are expected to become a new type of anti-tumor drug with high efficiency and low toxicity.

### Effects on the Cardiovascular System

Cardiovascular diseases refer to ischemic or hemorrhagic diseases in the heart, brain and whole body tissues caused by hyperlipidemia, blood viscous, atherosclerosis, and hypertension. *S. miltiorrhiza* and its compound preparations, such as composite Danshen droplet pills (Zhang et al., 2008) and Guanxin Danshen drop pills (Yao et al., 2018), have achieved good curative effects in the clinical treatment of cardiovascular diseases. Modern pharmacological research has found that *S. miltiorrhiza* has been widely used in the treatment of coronary artery, myocardial ischemia and myocardial infarction, improvement of microcirculation, and reduction of myocardial oxygen consumption.

Tanshinone IIA is a representative monomeric compound in *S. miltiorrhiza.* However, due to the poor water solubility, its water-soluble derivative, sodium tanshinone IIA silate (STS), is more widely used in the clinical treatment. Experimental results show that STS restrains the proliferation and migration of high glucose-induced vascular smooth muscle cells (VSMC), possibly through adenosine 5′-monophosphate (AMP)-activated protein kinase (AMPK) activation (Chen, 1984; Kyoko et al., 2002; Yang et al., 2007). In addition, STS has protective effects on myocardial apoptosis induced by acute myocardial infarction (MI) in adult rats (Yang et al., 2008) and inhibits cardiac fibrosis after pathological stimuli to the cardiovascular system (Le et al., 2009). The clinical efficacy results also show that tanshinone IIA sodium sulfonate injection is effective in the treatment of acute cerebral infarction, and can also be used as an additional treatment for patients with unstable angina pectoris (Zhang et al., 2014).

As the main lipophilic component of *S. miltiorrhiza*, tanshinone IIA also has cardiovascular protective effects. Its mechanisms of action mainly include the following: significantly reducing intimal thickening and restraining the proliferation and migration of VSMC, which is a significant event in the development of atherosclerosis (AS); by reducing the content of macrophages, reducing the uptake of oxidized low-density lipoprotein (ox-LDL) (Gong et al., 2009; Liu et al., 2014), promoting cholesterol excretion, and reducing the formation of foam cells, thereby regulating blood lipids and reducing body weight; inhibiting the development of left ventricular hypertrophy and reducing the level of apoptosis; significantly reducing blood viscosity, inhibiting thrombin activation, promoting fibrin degradation and inhibiting the formation of thrombosis (Wu L. et al., 2012). In summary, tanshinone IIA has broad application prospects in the prevention and treatment of cardiovascular diseases, but its specific mechanism remains to be further studied. In addition to tanshinone IIA, cryptotanshinone (Li et al., 2018), tanshinone I, dihydrotanshinone I (Wei Y. et al., 2016), etc., have proved to be effective coronary dilators, which can improve microcirculation and enhance vascular blood flow.

### Antioxidant Activity

Oxidative stress can cause DNA oxidative damage and abnormal expression of proteins puts the body in a vulnerable state, and is intently connected with the occurrence and development of various diseases. Previous studies have shown that tanshinone I, tanshinone IIB, cryptotanshinone, dihydrotanshinone I, methylenetanshinquinone, and miltiradiene (Houlihan et al., 1985) were all found to act as antioxidants (Ke et al., 1990; Weng and Gordon, 1992). The specific antioxidant activity of tanshinone IIA is that it can effectively restrain the interaction between DNA and the intracellular lipid peroxidation products (Cao et al., 1996; Niu et al., 2000; Zhou et al., 2013) and protect DNA by eliminating lipid free radicals and blocking the chain reaction of lipid peroxidation. In addition, tanshinone IIA inhibits atherosclerosis calcification in rats by inhibiting oxidative stress (Tang et al., 2007). Latter researches have indicated that it can also inhibit the activity and gene expression of nicotinamide adenine dinucleotide phosphate (NADPH) oxidase, as well as the production of reactive oxygen species (ROS) (Yang et al., 2010; Wang et al., 2011; Huang et al., 2018).

### Anti-bacterial Activity

The pharmacological effects of *S. miltiorrhiza* are very extensive, and in addition to the anti-tumor activity, which has been explored deeply, it has antibacterial and anti-inflammatory effects. The main lipophilic components (Lee et al., 1999) that play the antibacterial roles in *S. miltiorrhiza* are cryptotanshinone, dihydrotanshinone I (Honda et al., 1988), hydroxyl tanshinone and tanshinone IIB. These compounds could significantly inhibit gram-positive bacteria, especially cryptotanshinone and dihydrotanshinone I exhibited strong antimicrobial activity. They could bring forth superoxide radicals, which are of great significance in the antibacterial action. In comparison, tanshinone IIA and tanshinone I were confirmed to have medium antimicrobial activity (Zhao et al., 2011).

### Effects on Neurodegenerative Disorders

Neurodegenerative diseases are caused by the loss of neurons or their myelin, which over time, in succession causes degeneration of the structure and function of the central nervous system (Javed et al., 2016). Neurodegenerative diseases could be divided into two categories according to their phenotype: one that affects body movement, like Parkinson’s disease, and one that affects memory and related dementia, like Alzheimer’s disease (AD) (Prince et al., 2013; Ahmed et al., 2015; Orhan et al., 2015). Due to the good lipophilicity and small molecular weight, tanshinones are able to penetrate the blood-brain barrier (BBB). For example, tanshinone I (Jing et al., 2016), tanshinone IIA (Bi et al., 2008), and cryptotanshinone (Zhang F. et al., 2009; Park et al., 2012) have been reported to exhibit significant neuroprotective effects. Tanshinone I selectively suppressed pro-inflammatory gene expression in activated microglia and prevented nigrostriatal dopaminergic neurodegeneration in a mouse model of Parkinson’s disease (Wang S. et al., 2015). Tanshinone IIA protects neurons from the neurotoxicity of amyloid beta (Aβ) 25–35, increases neuronal viability and downregulates the expression of phosphorylated *tau* in AD. In addition, tanshinone IIA inhibits the transcription and expression of genes encoding inducible nitric oxide synthase (iNOS), metalloproteinase (MMP)-2, and human nuclear factor (NF-κBp65) pathway, thereby inhibiting the occurrence of neuroinflammation and reducing AD risk (Jiang P. et al., 2014).

### Other Bioactivities

In addition to the above mentioned pharmacological activities, tanshinone compounds exhibit anti-inflammatory effects (Fan et al., 2009), liver protection, protection against kidney damage, hormone-like activity, and so on. Methyl tanshinonate, hydroxytanshinone, tanshinone IIA, and tanshinone IIB show anti-platelet aggregation activity. Tanshinone IIA has the potential to ameliorate bone resorption diseases *in vivo* by reducing both the number and activity of osteoclasts (Han et al., 2007). Cryptotanshinone and dihydrotanshinone I have been reported to have anti-cholinesterase activity (Ren et al., 2004). Due to their wide range of pharmacological activities, tanshinones are expected to contribute to the development of new drugs for clinical treatment.

## Biosynthesis of Tanshinones in *S. miltiorrhiza*

**Figure 4:**
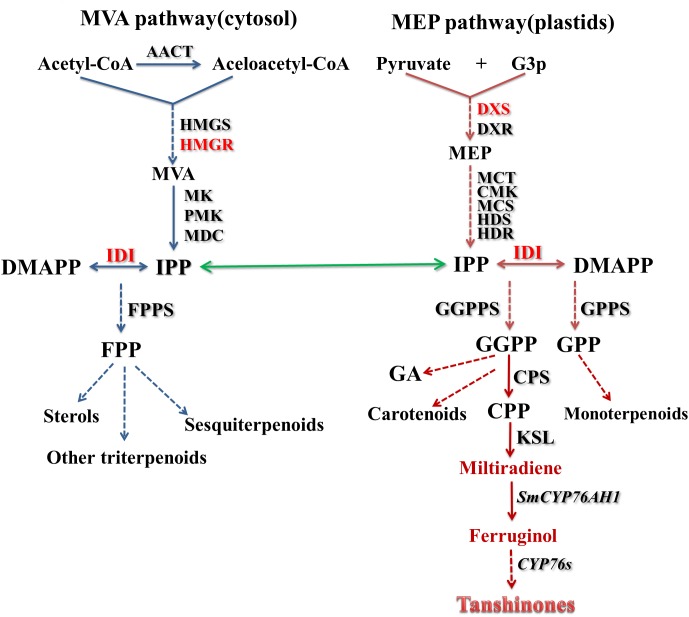
Outline of the biosynthesis pathway of tanshinones in *S. miltiorrhiza.* The common precursors IPP and DMAPP of the universal isoprene precursors are generated by MEP and MVA pathways. TPSs catalyze the production of GGPP by IPP and DMAPP, and GGPP further produces tanshinones through a series of cyclization and hydroxylation reactions. The abbreviations used are listed in Table 1.

Terpenoids are derived from methylpentahydroxy acid and have a molecular skeleton of an isoprene unit (C5 unit) as a basic structure unit. Terpenoids are widely found in nature and are also important class of compounds in Chinese herbal medicine (Cheng et al., 2007). According to the number of C5 units, terpenoids can be divided into monoterpenes, sesquiterpenes, diterpenes, and triterpenes, in addition to the more specific structure of iridoids. The biosynthesis pathway of terpenoids has certain rules to follow. The common precursor iospentenyl diphosphate (IPP) and its isomer dimethylallyl diphosphate (DMAPP) are generated by the 2-*C*-methyl-D-erythritol 4-phosphate (MEP) pathway and/or the mevalonate (MVA) pathway. Under the action of prenyltransferase, IPP and DMAPP condense to form precursors of monoterpenes, sesquiterpenes, diterpenes, and similar compounds. These precursors form the basic skeleton of various terpenoids under the action of a quinone synthase, and then form a final product under the action of a modifying enzyme such as cytochrome P450 hydroxylase or glycosyltransferase (Hasan et al., 2014; Zi et al., 2014). Tanshinones are a group of diterpenoids, and the biosynthesis pathway in *S. miltiorrhiza* also follows the above rules. Interference experiments have shown that the MVA pathway plays a more critical role in the cell growth of *S. miltiorrhiza* hairy roots, while the MEP pathway is more committed to the production of tanshinones (Yang et al., 2012). Of course, the interrelationship and crosstalk between the two pathways together promote cell growth and metabolite production (Figure 4).

### The MVA Pathway

Since the late 1950s, the MVA pathway has received extensive attention due to its involvement in the biosynthesis pathway of plant steroids. The MVA pathway begins with acetyl-CoA and ends with the production of IPP and DMAPP. 3-Hydroxy-3-methylglutaryl-CoA reductase (HMGR) (Dai et al., 2011), a rate-limiting enzyme in isoprenoid biosynthesis, catalyzes the conversion of HMG-CoA to MVA. There are four *SmHMGRs* (Cui et al., 2011) in the draft genome of *S. miltiorrhiza.*

The analysis of the tissue expression of the four genes is as follows: *SmHMGR1* has the highest tissue expression in flowers and root steles and the lowest expression in leaves; *SmHMGR2* is expressed in higher amounts in roots and stems; in addition to the low expression level in flowers, SmHMGR3 expressed the highest of the four homologous genes in other tissues (Zhang et al., 2012); in contrast, *SmHMGR4* has the highest tissue expression in flowers, but has the lowest expression in other tissues among the four homologous genes. Induced expression experiments show that *SmHMGR1, SmHMGR2* and *SmHMGR3* are sensitive to methyl jasmonate (MeJA) (Pan et al., 2009; Dai et al., 2011; Ma et al., 2012). Overexpression of the above three genes can significantly increase the content of tanshinones in hairy root culture.

In addition to *SmHMGR*s, the genes involved in the MVA pathway include *SmAACT, SmHMGS*, *SmMK*, *SmPMK*, and *SmMDC* (Cui et al., 2010; Lin et al., 2011; Ma et al., 2012). The proteins encoded by these genes each play a specific function, catalyzing the corresponding reactions and performing their duties; thus, IPP and DMAPP are finally obtained from the MVA pathway.

### The MEP Pathway

The MEP pathway, also appearing the non-mevalonate pathway, uses pyruvate (Pyr) and glyceraldehyde 3-phosphate (G3P) as precursors to produce the two products (IPP and DMAPP) under the action of a series of enzymes. In plants, the MEP pathway is localized in the plastid and provides precursors of the synthesis of terpenoids, as well as side chains of chlorophylls and tocopherols.

Many genes in the MEP pathway have been cloned and identified (Wang et al., 2008; Gao W. et al., 2012; Ma et al., 2012; Cheng Q.Q. et al., 2013; Hao et al., 2013; Jiang D. et al., 2014). 1-Deoxy-D-xylulose-5-phosphate synthase (DXS) is the first enzyme in the MEP pathway and a key regulatory site for the pathway (Wei Z. et al., 2016). There are five DXSs (Ma et al., 2012) in the draft genome of *S. miltiorrhiza*, and *SmDXS2* expression was predominantly detected in roots than the other four (Kai et al., 2011; Wu et al., 2011), which suggested that *SmDXS2* is more likely to be involved in the biosynthetic pathway of tanshinones. Phylogenetic analysis showed that *SmDXS2* and *SmDXS3* were clustered into one branch, belonging to the DXS2 clade. *SmDXS3* also has high tissue expression in roots and root steles and is highly sensitive to MeJA, indicating that it may be involved in plant defense mechanisms.

*SmDXR* catalyzes 1-deoxy-D-xylulose 5- phosphate reductoisomerase (DXP) to generate MEP. *SmDXR* is expressed in various tissues and has the highest expression in roots. Under high-osmotic stress and yeast elicitors, the content of tanshinones in *S. miltiorrhiza* hairy roots increased significantly and RT-PCR results showed that the transcription level of *SmDXR* gene was also upregulated. The DXR genes may contribute to the control of tanshinone metabolic route and be a regulatory site for efficient heterogeneous production of tanshinones (Yan et al., 2009; Kai et al., 2011; Wu et al., 2011).

### Genes Involved in the Miltiradiene Branch Pathway

Isopentenyl diphosphate isomerase (IDI), also known as isopentenyl pyrophosphate isomerase, catalyzes the conversion of the relatively unreactive IPP to the more-reactive electrophile DMAPP. Two IDI genes (*SmIDI1* and *SmIDI2*) have been found and cloned from *S. miltiorrhiza*. *SmIDI1* has been confirmed as a candidate gene involved in the biosynthesis of tanshinone (Cui et al., 2011; Yang et al., 2011). Tissue expression analysis revealed that *SmIDI1* has the higher expression in leaves than in the roots and stems. The relative expression of *SmIDI1* increased under the induction of Ag^+^ and MeJA. Color complementation experiments showed that *SmIDI1* promoted the accumulation of lycopene. Compared with *SmIDI1*, there are few studies about *SmIDI2* (Ma et al., 2012), which has a chloroplast-localized transit peptide, whereas *SmIDI1* does not have one. The tissue expression level of *SmIDI2* was lower than that of *SmIDI1* in each analyzed tissue. In general, *SmIDI1* may play a more important role in the biosynthesis process of tanshinones.

As universal precursors for the biosynthesis of terpeniods, both IPP and DMAPP condense to form precursors for monoterpene, diterpene, sesquiterpene and triterpenes, and so forth under the action of specific prenyltransferases. For example, one molecule of IPP and one molecule of DMAPP generate farnesyl diphosphate (FPP) under the catalysis of farnesyl diphosphate synthase (FPPS). FPPS belongs to family of short-chain prenyltransferases which also includes geranyl diphosphate synthase (GPPS), geranylgeranyl diphosphate synthase (GGPPS).

Analysis of the *S. miltiorrhiza* genome shows there are five GPPS genes (*SmGPPS* encoding the homomeric GPPS subunit; *SmGPPS.LSU*, *SmGPPS.SSUI*, *SmGPPS.SSUII.1*, and *SmGPPS.SSUII.2* encoding heteromeric GPPS subunits), one FPPS gene (*SmFPPS*), and three GGPPS genes (*SmGGPPS1*, *SmGGPPS2*, and *SmGGPPS3*) (Cui et al., 2011; Kai et al., 2011; Ma et al., 2012, 2015).

GPPS is generally considered to be regarded as the backbone of monoterpene constituents, but recent studies have found that it is also necessary in the biosynthesis pathway of some diterpenoids, such as gibberellins (van Schie et al., 2010). To date, there are still few studies on the five GPPS genes in *S. miltiorrhiza*. The differences in homology and tissue specificity also make the research more difficult. The monoterpenes and sesquiterpenoids contained in many *Salvia* species are mainly enriched in the trichomes of the aerial parts. They also have a wide range of pharmacological activities, such as antimicrobial and antioxidant activities. There is still a need to further identify and characterize the *SmFPPS* and *SmGPPS* genes to explore the roles they played in the biosynthesis of essential oils in *S. miltiorrhiza* (Ma et al., 2012).

GGPPS catalyzes the condensation reaction between FPP and IPP to form GGPP. GGPP is a key precursor of biosynthesis of diterpenoids, chlorophylls, and carotenoids. *S. miltiorrhiza* contains three GGPPS genes. The full-length cDNA of *SmGGPPS1* has been isolated by rapid amplification of cDNA ends (RACE). Tissue expression pattern analysis showed that *SmGGPPS1* expression was higher in leaves and roots than stems. Functional identification experiments showed that *SmGGPPS1* can catalyze the production of carotenoids, and the introduction of *SmGGPPS1* gene into the transgenic hairy roots could significantly increase the content of tanshinones. So far, there are few studies on *SmGGPPS2* and *SmGGPPS*3, the functions of the two genes remain to be elucidated (Zhang L. et al., 2009; Kai et al., 2010; Hua et al., 2012; Ma et al., 2012; Yan et al., 2014).

Comparing the structures of GGPP and tanshinone compounds, the differences can be clearly observed and GGPP needs to undergo a cyclization to form tanshinones. The key enzymes involved in the process from GGPP to the production of miltiradiene are labdadienyl/copalyl diphosphate synthases (CPS) and kaurene synthase-like (KSL), which are class II diterpene cyclases. There are twelve *SmCPS* and nine *SmKSL* homologs reported in *S. miltiorrhiza* and based on the genome sequences (Ma et al., 2012), seven cDNA sequences encoding TPSs (including *SmCPS1* to *SmCPS5*, *SmKSL1*, and *SmKSL2*) have been cloned. Previous work has indicated that *SmCPS1* and *SmKSL1* participate in the tanshinone biosynthesis (Gao et al., 2009) and using the modular path engineering (MOPE) strategy, *SmCPS*, *SmKSL* and other related genes construction fusion proteins were expressed in the optimized yeast strain and finally the yield of miltiradiene in the 15 L bioreactor reached 365 mg/L (Zhou et al., 2012). Combining *SmCPS2* and *SmKSL1* expressed proteins and feeding GGPP as substrate also lead to production of miltiradiene. Phylogenetic analysis indicates that *SmCPS1* to *SmCPS3* are closely related to CPSs involved in the biosynthesis of CPP, while *SmCPS4* and *SmCPS5* are closely related to the CPSs generally for gibberellin biosynthesis (Ma et al., 2012; Cheng Q. et al., 2013; Cui et al., 2015). Compared with *SmKSL2*, *SmKSL1* has experienced a loss of the N-terminal g-domain, which indicates that *SmKSL1* involved in a more specific diterpenoid metabolic process. Phylogenetic analysis also indicated *SmKSL2* clusters with other dicotyledonous KSL genes, many of which have been identified to be involved in GA biosynthesis (Gao et al., 2009; Ma et al., 2012; Cui et al., 2015).

Under the action of TPS, GGPP undergoes cyclization reactions to form miltiradiene, which needs to be further modified to produce tanshinones. These modification reactions include decarboxylation, oxidation and reductions, and each step can be catalyzed by the corresponding specific enzyme.

Cytochromes P450 is an important mixed-function heme oxidoreductase in animals, plants and microorganisms, and is a gene superfamily (Ghosh, 2017). It can catalyze a variety of chemical reactions and plays an important role in protecting organisms from external aggression and in plant secondary metabolism (David and Danièle, 2011). CYP450s often form multi-enzyme complexes with other enzymes on the endoplasmic reticulum to act on the biosynthetic pathway of phenylpropanoid. With the development of modern sequencing technology and the analysis of plant genomes, more and more P450 genes involved in plant secondary metabolic activities have been discovered (Chen et al., 2014). The transcripts of the induction process of *S. miltiorrhiza* hair roots were analyzed to reveal 125 CYPs expressed therein (Gao et al., 2014). Guo et al. used a next-generation sequencing approach to identify six candidate CYP genes being related with the biosynthesis of tanshinones in both the rhizome and Danshen hairy roots. Among these six genes, they identified *SmCYP76AH1* could catalyze the conversion of miltiradiene to ferruginol *in vivo* and *in vitro*, making the biosynthesis pathway of tanshinone a critical step. And the ferruginol content reached 10.5 mg/L by heterologous expression of *SmCYP76AH1* and *phyto-*CYP reductase genes in yeast (Guo et al., 2013). With further research, they found other two *SmCYP*s, respectively, named *CYP76AH3* and *CYP76AK1*, exhibiting similar transcription profiles as *SmCYP76AH1* in elicited *S. miltiorrhiza* hairy roots (Guo et al., 2016). *SmCYP76AH3* oxidizes ferruginol at two different carbon centers to obtain 11-hydroxy ferruginol and 11-hydroxy sugiol. And then *SmCYP76AK1* could hydroxylate C-20 of two of the resulting intermediates. Together, sequential cooperation between two CYPs convert ferruginol into 11, 20-dihydroxy ferruginol and 11, 20-dihydroxy sugiol which may be a bifurcating pathway for the biosynthesis of tanshinones. After analyzing the structure of ferruginol and tanshinones, a putative biosynthetic pathway can be inferred, that is, ferruginol can be dehydrogenated to form cryptotanshinone, and then cryptotanshinone can be reduced by reductases to form tanshinone IIA (Cai et al., 2013; Guo et al., 2016).

Two full length cytochrome P450 reductase (CPR) genes have also been cloned from *S. miltiorrhiza* named *SmCPR1* and *SmCPR2*. Although there are relatively few studies on these two genes, they have a great role in supporting the CYP450 genes to play the catalytic functions (Schenkman and Ingela, 2003). The experiment shows that when importing *SmCYP76AH1* alone into the miltiradiene-producing yeast strain failed to produce ferruginol, in contrast yeast strains with plant CPRs after introducing *SmCYP76AH1* could produce ferruginol (Zhou et al., 2015).

The biosynthetic pathway from ferruginol to tanshinones remains to be further resolved. The discovery and comparison of structural features of intermediates that may occur in the tanshinone biosynthesis pathway represent a more effective way to find related functional enzymes, excavate functional genes, and analyze the complete pathway. Currently, the field of gene sequencing is full of unknowns and expectations. The development and application of fourth-generation gene sequencing technology will definitely bring brighter prospects to the fields of scientific research and medicine.

## Synthetic Biology of Tanshinones

In recent years, the discovery of key genes for the biosynthesis of medicinal active ingredients and the use of synthetic biology strategies to design and engineer microbial strains to produce natural products have been considered a promising method for resource acquisition (Gao W. et al., 2015). Miltiradiene is a key precursor for the biosynthesis of tanshinones. At present, *Saccharomyces cerevisiae* is used as the chassis cell, the engineered strains with a high yield of miltiradiene were constructed by designing functional modules; the yield could reach 488 mg/L (Gao et al., 2009; Zhou et al., 2012). The cloning of the first modified enzyme gene *CYP76AH1* downstream of the tanshinone biosynthesis pathway successfully transformed miltiradiene into ferruginol, which is stable and biologically active. The highest yield of ferruginol reached 10.5 mg/L under shake flask fermentation conditions (Guo et al., 2016).

Increasingly more scholars have focused on not only functional genes in the biosynthetic pathway but also the effects of elicitors and regulatory genes on secondary metabolites in plants. Elicitors can directly or indirectly prompt the accumulation of plant secondary metabolites in plant tissues, cell culture and hairy root culture. According to their sources, these elicitors can be divided into biological inducers and abiotic inducers. Biological elicitors such as *Trichoderma atroviride* (Ming et al., 2013), *Chaetomium globosum* D38 (Zhai et al., 2017), *Alternaria sp.* A13 (Zhou et al., 2018) and other endophytic fungi, as well as some non-biological inducers such as MeJA, salicylic acid, yeast extract, Ag^+^ (Xing et al., 2014), and ultraviolet-B (Wang et al., 2016), could induce the accumulation of active ingredients in *S. miltiorrhiza*.

## Conclusion and Prospects

As a perennial cross-pollinating plant species, *S. miltiorrhiza* is strongly adaptive and widely distributed with generations having overlapping features and significant genetic diversity. Since ancient times, the single-component Danshen or its combination with other drugs has been used in the clinical treatment of various diseases exhibiting good effects. *S. miltiorrhiza* contains a variety of active ingredients, among which tanshinones have exhibited significant pharmacological properties, such as antibacterial, antioxidant, anti-inflammatory, and anti-tumor activities. In this paper, the extraction and purification methods of tanshinones are briefly introduced. Many new methods and new technologies have been applied, and it is worth mentioning that the combination of different methods can achieve high efficiency and low consumption and may have promising development prospects.

The traditional method of obtaining tanshinones has been unable to meet the clinical drug needs. In recent years by continuously investigating the functional genes in the tanshinone biosynthesis pathway, analyzing the complete pathway and reconstituting the biosynthetic pathway in heterologous hosts, the method of efficiently and heterologously producing tanshinones has become the focus of attention. To date, studies have shown that miltiradiene is an intermediate for the biosynthesis of tanshinones, and that it can be converted to tanshinones under the action of various modified enzymes. Therefore, more functional genes must be further discovered. Synthetic biology strategies and methods not only provide ideas for more efficient and convenient access to tanshinones, but also lay the foundation for the sustainable use of traditional Chinese medicine resources.

## Author Contributions

ZJ, WG, and LH contributed the conception and design of the study. ZJ organized the database and wrote the first draft of the manuscript. WG revised parts of the manuscript. All authors contributed to manuscript revision, read and approved the submitted version.

## Conflict of Interest Statement

The authors declare that the research was conducted in the absence of any commercial or financial relationships that could be construed as a potential conflict of interest.
